# Transanal endoscopic microsurgery in treatment of rectal adenomas and T1 low-risk carcinomas

**DOI:** 10.1186/1477-7819-10-255

**Published:** 2012-11-26

**Authors:** Michael Amann, Ali Modabber, Jens Burghardt, Christian Stratz, Claudius Falch, Gerhard F Buess, Andreas Kirschniak

**Affiliations:** 1 Department of Cardiology, Bad Krozingen Heart Center, Südring 15, 79189, Bad Krozingen, Germany; 2 Department of Oral, Maxillofacial and Plastic Facial Surgery, University Hospital Aachen, Pauwelsstraße 30, 52074, Aachen, Germany; 3 Department of Surgery, Freikirchliches Krankenhaus und Poliklinik Rüdersdorf, Seebad 82/83, 15562, Rüdersdorf, Germany; 4 Department of General, Visceral and Transplant Surgery, Tübingen University Hospital, Hoppe-Seyler-Straße 3, 72076, Tübingen, Germany

**Keywords:** Transanal endoscopic microsurgery, Rectal adenoma, Rectal carcinoma, Local excision, Endoscopic surgery

## Abstract

**Background:**

Transanal endoscopic microsurgery as a local therapy option for rectal neoplasms is a tissue-sparing technique that protects the anal sphincter. The present retrospective analysis reports the course of observation after local excision of adenomas and T1 low-risk carcinomas using transanal endoscopic microsurgery.

**Methods:**

In a retrospective analysis we examined data on 279 patients for local recurrence. A total of 144 patients had a rectal adenoma (*n* = 103) or a R0 resection of low-risk T1 carcinomas (*n* = 41). In this collective, we also examined parameters concerning perioperative management, complications, intraoperative blood loss and duration of hospital stay.

**Results:**

Patients with adenoma were on average 64.9 (range 37 to 90) years old; 83.5% of the adenomas were located 3 to 11 cm from the anocutaneous line. In adenoma patients the recurrence rate was 2.9% for an observation period of 21.8 months. The postoperative course was without any complications in 98.1% of patients.

Patients with T1 low-risk carcinoma were 64.6 (range 30 to 89) years old. In all cases, an R0 resection could be performed. The recurrence rate was 9.8% for an observation period of 34.4 months. In this group the postoperative course was free of complications in 97.6% of patients.

**Conclusions:**

The high efficacy of transanal endoscopic microsurgery ensures minimally invasive treatment of adenomas and low-risk T1 carcinomas with low complication rates and a low rate of therapeutic failure.

## Background

The incidence rate of colorectal cancer in Europe is about 0.00025% a year; 29% of these carcinomas are located in the rectum [1].

Autopsy studies reveal that 34 to 36.9% of men and 28.7 to 32% of women have rectal adenomas [2,3]. Technological progress offers new possibilities for the surgical treatment of rectal neoplasms. There are different local excision techniques such as the methods of Mason and Parks [4,5]. Also, endoscopic polypectomy or endoscopic submucosal dissection (ESD) is used in clinical daily routine with a high rate of *en bloc* resection and curative rates [6-8]. ESD offers a newer, safe opportunity in the surgical treatment of tumors with diameter >20 mm. The high rate of perforations can be managed in most cases by conservative endoscopic treatment [8]. Several studies have shown that transanal endoscopic microsurgery (TEM) is a very suitable method for surgical treatment of broad-based adenomas [9-13].

The literature reports the recurrence rate for transanal polypectomy using Parks’ method to be between 16 and 20% [14,15]. In 5% a revision operation is necessary because of complications [16]. A recrudescence rate of 3.3% is reported for the Mason method [14], with a morbidity rate of 30.7% because of fistulas and bleeding [17]. However, a high degree of wound infections, fistulas and a high mortality rate are reported for these methods [18]. A comparative study showed TEM to be less invasive and to entail lower complication rates and shorter duration of hospital stay than the Mason method [19]. Moreover, a reduced complication and recurrence rate is reported for TEM as compared with direct local excision [20]. Last but not least, TEM is significantly less expensive than local excision or anterior resection of adenoma [20].

The literature on treatment of T1 carcinomas with low-risk histology states that when taking account of all relevant factors such as treatment costs, complication rates and mortality, TEM with continuous follow-up examinations is superior to the radical methods [19,21]. An important factor in T1 carcinomas is the differentiation between low-risk and high-risk histology. Nowadays tumors are classified in sm-stadiums sm1 to sm3 according to their depth of submucosal penetration. Patients with sm2 to sm3 are high-risk cases for treatment with TEM alone [22]. Our patient collective dates from a time before this classification were established. Our collective, called T1 low-risk carcinomas, includes T1 carcinomas with histological grading G1 to G2, R0 resection and the absence of microlymphatic or microvascular invasion.

The aim of this retrospective analysis is to show that TEM is a safe, minimally invasive technique with a low recurrence rate for the treatment of rectal adenomas and T1 carcinomas with low-risk histology (histological grading G1 to G2, R0 resection and the absence of microlymphatic or microvascular invasion).

## Patients and methods

### Patients

Overall 279 patients with rectal neoplasm had a local excision with TEM in our sections in the Olympiapark-Klinik Munich and the Helios-Klinik Müllheim between 1998 and 2006. The patients signed written informed consent forms concerning the potential surgical risks. Out of this group, data for 144 patients with rectal adenoma (*n* = 103) and low-risk T1 carcinoma (*n* = 41) were retrospectively evaluated for local recurrence. The rest of our collective (*n* = 135) had advanced stages of rectal carcinomas. These patients were treated by TEM because of multimorbidity, older age or because they avoided a radical surgical procedure. This subgroup is excluded from the present study.

The primary endpoint of the study was the recurrence rate of adenoma or carcinoma in all study patients who underwent continuous follow-up examinations including, among others, rectoscopy and endoscopic ultrasonography.

The collected data were analyzed with the database management system Microsoft Access 2003 (Microsoft Corporation, Redmond, WA, USA). The database documented tumor characteristics such as size, pathology and distance from the anocutaneous line. We collected data on the applied excision method, intraoperative blood loss, operation time, perioperative complications and duration of hospital stay.

### Surgical procedure

Before surgery, physical examination with digital rectal palpation, rectoscopy and endosonography for the localization and extent of the tumor were performed in all patients. In addition, preoperative histology with biopsy forceps was performed.

Patients with adenomas and T1 low–risk histology (pathologic findings (G1 to G2), R0 resection and the absence of microlymphatic or microvascular invasion) were included.

All patients underwent surgery using the TEM technique, which was described for the first time by Buess and colleagues [9,23]. Surgery was performed on the operating table using a fixed, wide-lumen rectoscope. Complex microsurgical instruments including lenses providing a three-dimensional view were introduced through the rectoscope. The TEM instrument set used was obtained from Richard Wolf GmbH (Knittlingen, Germany). A full-thickness intestinal wall excision was performed from 3 to 11 cm from the anocutaneous line in the dorsal area. Because of the risk of perforation, a partial-thickness excision of the intestinal wall was performed if the tumor extended to the intraperitoneal region.

## Results

### Adenomas

The group of patients with adenoma included 103 patients, 57 patients were female and 46 male. The mean age was 64.9 (range 37 to 90) years, median 65 years. Of the patients, 83.5% preoperatively reported being symptom free, 9.7% consulted a physician because of bloody stools, 4.9% because of bloody mucus, while 1.9% had other symptoms.

**Figure 1:**
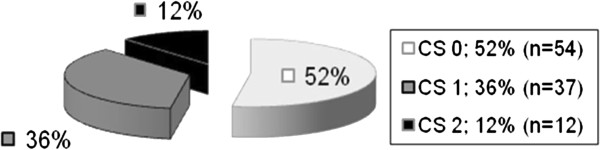
Clinical staging of the preoperatively performed digital examination in the adenoma group.

**Figure 2:**
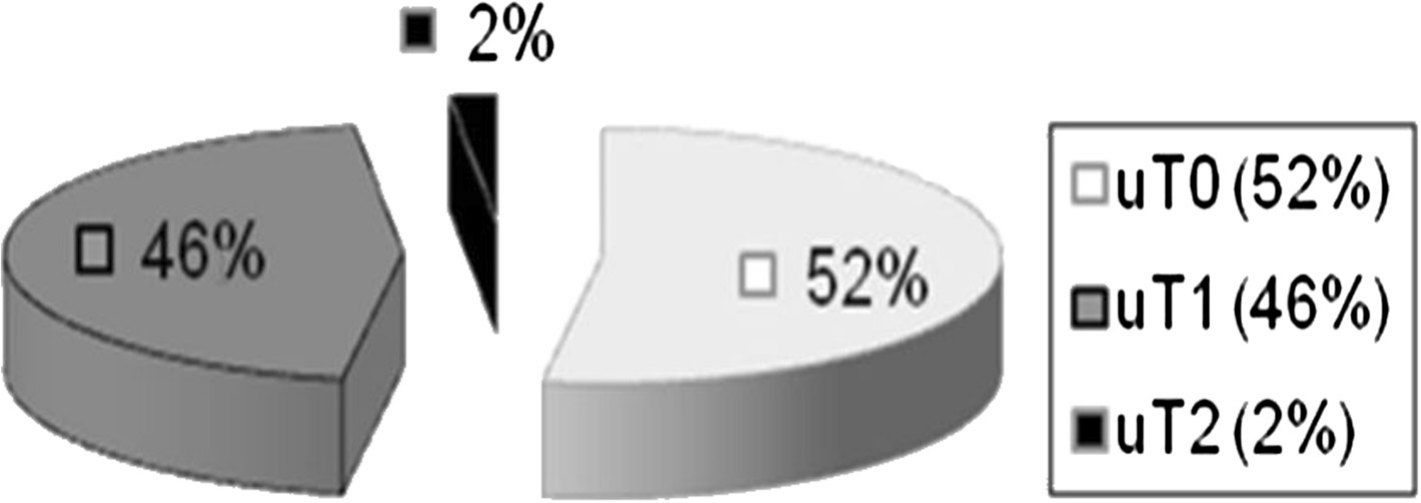
Results of the endosonographic ultrasound examination in the adenoma group.

Digital rectal palpation was classified according to the modified clinical staging of Mason [24] (Table 1). The results of digital examination are shown in Figure 1 and the results of preoperatively performed endosonographic findings in Figure 2.

**Table 1:** Clinical staging of Mason

**Stage**	**Definition**	**Pathological correlation**
Clinical Stage 0	No rough parts of tumor	
Clinical Stage I	Freely mobile	Submucosa
Clinical Stage II	Mobile	Muscularis propria
Clinical Stage III	Tethered mobility	Perirectal fat
Clinical Stage IV	Fixed/tethered fixation	Adjacent structures

**Figure 3:**
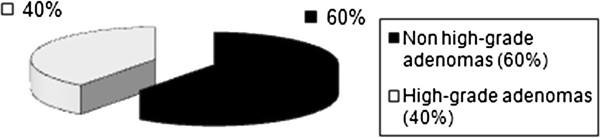
Preoperative histological findings in the adenoma group.

Preoperative histological findings showed in 60% of the study patients a nonhigh-grade adenoma, and in 40% a high-grade adenoma (Figure 3). In the final histological evaluation following TEM, 51 patients had a nonhigh-grade adenoma and 52 patients a high-grade adenoma.

Tumor distance was measured from the lower rim of the tumor to the anocutaneous line. Of the adenomas 83.5% were located 3 to 11 cm from the anocutaneous line, while most (37.9%) of them were at a distance of 6 to 8 cm and 16.5% were situated >11 cm from the anocutaneous line.

Full-thickness excision of the intestinal wall was performed in 87.4% of patients, while 12.6% of the patients underwent partial-thickness excision of the intestinal wall to avoid perforation to the abdominal cavity. Intraoperative blood loss averaged 20.6 (range 5 to 100) ml. In the adenoma group we had no severe intraoperative complications such as perforation or uncontrolled bleeding.

The postoperative course was without complications in 101 (98.1%) patients. In two cases a rectal stenosis was postoperatively observed by endoscopy; it was overcome with a rectoscope and treated with stool-regulatory measures. Duration of hospital stay after surgery was on average 8.8 (range 1 to 18) days. Twenty-four of 103 patients (23.3%) had a tumor size ≤3 cm. Fifty-four patients (52.4%) had a tumor diameter >3 cm, with a maximum of 5 cm. A tumor diameter >5 cm with a maximum of 7 cm occurred in 17 patients (16.5%). In eight patients the tumor diameter was even larger than 7 cm.

**Figure 4:**
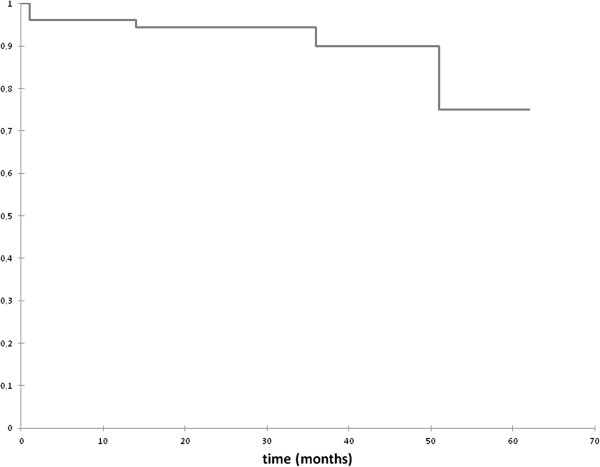
**Kaplan**–**Meier curve for the probability of relapse**-**free time of adenomas.**

After a follow-up period of 21.8 months, 96 (93.2%) patients were free of recurrence with a follow-up rate of 96.1% (Table 2). Three patients relapsed after local excision with TEM. All of them had a re-TEM and they were relapse free until the end of the observation period. Figure 4 illustrates the Kaplan–Meier curve for the probability of relapse-free time.

**Table 2:** **Follow**-**up of the adenoma group**

	**Nonhigh-grade**	**High-grade**	**Overall**
*n*	51	52	103
Recurrence free (*n*)	47	49	96
Lost to follow-up (*n*)	1	3	4
Recurrences (*n*)	3	0	3
Mean relapse-free survival (months)	19.4	22.9	21.1
Mean duration of observation period (months)	20.7	22.9	21.8

### T1 low-risk carcinomas

The group of patients with low-risk T1 carcinoma included 41 patients (16 female, 25 male). The mean age was 64.6 (range 30 to 89) years, median 65 years.

**Figure 5:**
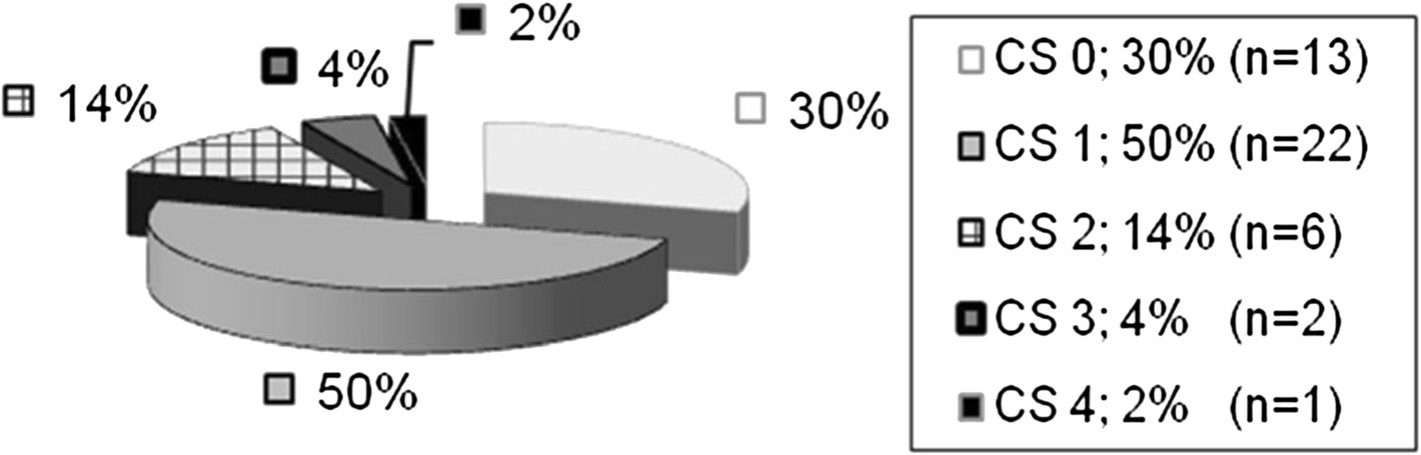
Clinical staging of the preoperatively performed digital examination in the T1 carcinoma group.

**Figure 6:**
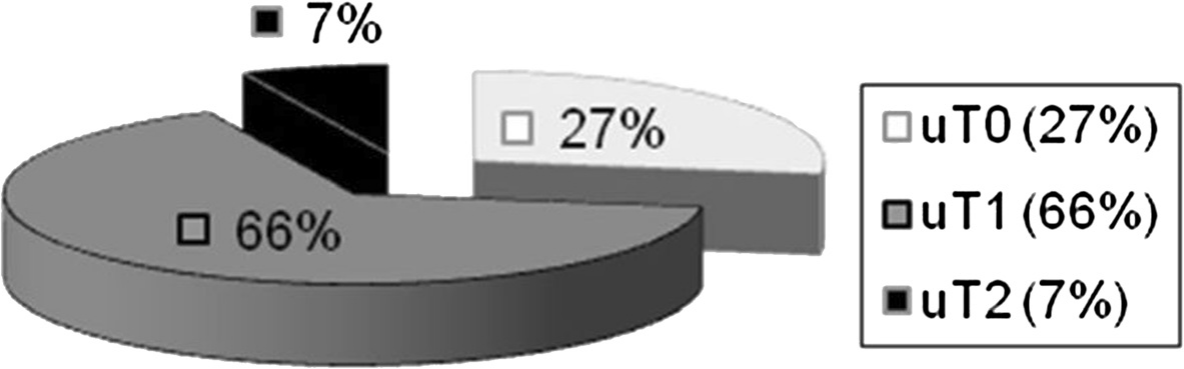
Results of the endosonographic ultrasound examination in the T1 carcinoma group.

**Figure 7:**
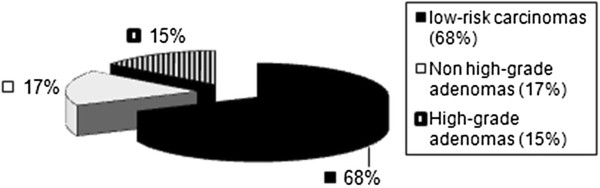
Preoperative histological findings in the T1 carcinoma group.

Before surgery 62.1% of the patients were symptom free, 23.3% reported bloody stools and 6.8% mucus as the initial symptom, while 7.8% indicated other symptoms such as stool irregularity. The results of digital rectal palpation according to the Clinical Staging of Mason are shown in Figure 5. Preoperative endosonographic and histological findings are shown in Figures 6 and 7.

In 13 of 41 T1 low-risk carcinoma findings, preoperative histological examination showed adenomas (32.0%). Consistent with preoperative histological findings, 28 (68.0%) patients had a T1 low-risk carcinoma. As in patients with adenoma, 83.3% of T1 carcinomas were located 3 to 11 cm from the anocutaneous line, whereby most (39.8%) of the carcinomas were at a distance of 6 to 8 cm; 16.7% were more than 11 cm from the anocutaneous line.

In 89.3% of our cases a full-thickness excision of the intestinal wall was performed, and 10.7% of the study patients were treated with a partial-thickness excision of the intestinal wall.

Average intraoperative blood loss was 57.7 ml, median 10 (range 5 to 1,500) ml. Most of the patients (40 of 41 patients) had no severe intraoperative complications. In one patient, uncontrolled intraoperative bleeding caused delayed hemostasis and blood transfusion was necessary.

The postoperative course was without complication in 40 out of 41 patients. In one patient, a suture insufficiency occurred during hospitalization. This was diagnosed by control rectoscopy and treated conservatively. Postoperative hospital stay averaged 10.0 (range 4 to 23) days.

In seven out of 41 patients (17.1%) the tumor size was ≤3 cm. Twenty-three of 41 patients (56.1%) had a tumor size >3 cm with a maximum of 5 cm. Eleven patients (26.8%) even had a tumor size larger than 5 cm.

Therapy consisted exclusively of local excision by means of TEM. In all 41 patients an R0 resection was performed; 35 of these patients underwent regular follow-up without recurrence or complaints. Histological findings showed T1 low-risk carcinomas (G1 to G2, L0, V0) with a follow-up rate of 95.1%. Only two patients were lost to follow-up. Thirty-five (85.4%) patients were recurrence free.

Four patients relapsed after 13 to 71 months (Table 3). All of them had an initial tumor size >3 cm and two of them had a partial-thickness excision.

**Table 3:** **Follow**-**up of the T1 low**-**risk carcinoma group**

	**T1 low-risk carcinomas**
*n*	41
Recurrence free (*n*)	35
Lost to follow-up	2
Recurrences (*n*)	4
Mean relapse-free survival (months)	29.5
Mean duration of observation period (months)	34.4

Two patients were given adjuvant radiochemotherapy and re-TEM, subsequently remaining relapse-free for 35 and 39 months, respectively. The third patient relapsed after 30 months and was operated with curative intent using a deep anterior rectal resection to preserve the sphincter. This was followed by adjuvant radiochemotherapy. In the fourth patient, relapse was diagnosed 71 months after carcinoma excision by means of TEM, local recurrence and liver metastases. The patient subsequently underwent palliative radiochemotherapy.

Average relapse-free survival was 29.5 months with a median of 25 months. Average duration of the observation period was 34.4 months, median 27 months.

The overall recurrence rate in patients with T1 low-risk carcinoma was 9.8%. Of the relapsed patients, 75% underwent curative treatment.

**Figure 8:**
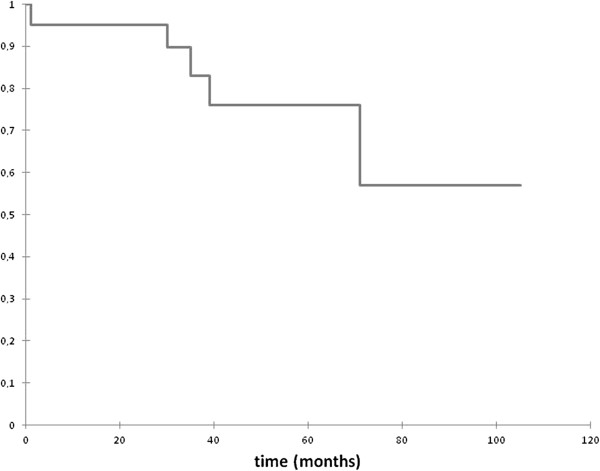
**Kaplan**–**Meier curve for the probability of relapse**-**free time of T1 low**-**risk carcinomas.**

The rate of failure was thus 2.4%. Figure 8 shows the Kaplan–Meier curve for the probability of relapse-free time.

## Discussion

The study collective was analyzed retrospectively. Patients with histologically determined adenomas and T1 low-risk carcinomas were included. When planning optimal treatment of rectal neoplasms, preoperative assessment of tumor extension should be as accurate as possible. All patients in this study collective underwent rectoscopy, digital rectal examination, transrectal endosonography and biopsy, among other diagnostic procedures.

Adenomas were retrospectively classified as uT0 in 52% of patients, uT1 in 46%, and uT2 in 2% (Figure 2). On the other hand, T1 low-risk carcinomas were classified as uT0 in 27% of patients, uT1 in 66% and uT2 in 7% (Figure 6). Ashraf and colleagues reported the accuracy of endorectal ultrasound to be disappointing for preoperative staging [25]. Zorcolo and colleagues also confirmed that differentiation between T0 and T1 with endorectal ultrasound remains a challenge, even though it does not influence surgical strategy [26].

In the group of patients with adenoma, preoperative histology already pointed to an adenoma in each patient (Figure 3). In this study the preoperative histology of patients in the T1 low-risk carcinoma group already showed a low-risk carcinoma in 68.0% of patients. In 32% the biopsy showed an adenoma (Figure 7). This confirms the limited accuracy of preoperative biopsy, as mentioned in the literature [27].

Transrectal ultrasound showed stage uT0/uT1 in 101 (98.1%) of the 103 patients in the adenoma group. Digital rectal palpation classified 52.4% (54/103) of the patients as Clinical Stage 0 and another 35.9% (37/103) as Clinical Stage 1. The biopsy for each patient showed an adenoma.

For T1 low-risk carcinomas, digital–rectal palpation showed a Clinical Stage 1 carcinoma in 50.5% of patients. Transrectal ultrasound showed stage uT0/uT1 in 38/41 (92.%) patients. As mentioned, preoperative histology showed a low-risk carcinoma in 68% of patients.

Two percent of adenomas and 7% of carcinomas were tagged uT2 at the preoperative endosonographic examination. TEM was offered to these patients as a curative therapeutic option because there is no statistical difference in the 5-year survival rate between TEM and radical surgery for T2 carcinomas [28].

Once more we therefore see that only a combination of examinations can provide the most accurate tumor assessment. This information can be used for surgical planning and for the differentiation between adenomas and T1 low-risk carcinomas. Our study population showed a perioperative mortality rate and a rate of ostomy pouch of 0%. These known advantages of TEM, particularly as compared with the radical surgical procedure, were confirmed by our study [29]. Moreover, this study performed in a larger collective confirms that TEM-induced lesser blood loss decreases postoperative risk [12] and also confirms the shortened duration of hospital stay following local excision [30]. This is very important, especially from a financial point of view. In our collective the average hospital stay is long compared with the hospital stay after TEM. Some of our patients had local excision in 1998 when our experiences about TEM and postoperative complications were lower. For example, in 2006 our average hospital stay was much shorter compared with 1998 (5.7 days vs. 11.8 days).

The 103 adenoma patients showed a recurrence rate of 2.9% in an average observation period of 21.8 months. This correlates with data reported to date in the literature, namely recurrence rates of 2.2 to 16% for adenomas following local excision. TEM is therefore described as an appropriate method for treating broad-based rectal adenomas [10-12]; the results of our study confirm this suggestion.

A novel procedure that also enables resection of early rectal neoplasm is ESD. Some comparative studies have shown that ESD offers better short-term clinical outcomes with shorter hospital stay than a local excision approach [6,31]. Nevertheless, larger studies are needed to validate the clinical long-term outcome. A comparative study concerning the effectiveness of ESD with conventional endoscopic mucosal resection for tumors >20 mm diameter reveals that ESD has a longer procedure time and higher perforation rate but higher *en bloc* resection and curative rates compared with the conventional endoscopic mucosal resection [7,8]. By conservative endoscopic treatment, the perforations after ESD could be managed [8]. The role of TEM in the treatment of tumors with diameter >20 mm therefore nowadays has minor clinical importance since the introduction of ESD into the clinical daily routine.

The superiority of TEM versus total mesorectal excision in the treatment of T1 carcinomas is the subject of debate in the literature. Lezoche and colleagues reported that long-term results after local excision using TEM are not inferior to those reported for total mesorectal excision [32]. A comparative study for T1 carcinomas showed TEM to be much safer, survival being comparable with that of total mesorectal excision [33].

In our sample the recurrence rate for patients with T1 low-risk carcinoma was 9.8% for an average observation period of 34.4 months. A limitation of this study is the fact that the depth of submucosal penetration of T1 low-risk carcinoma was not determined in our collective, because it is an important prognostic risk factor for recurrence [22]. Doornebosch and colleagues showed that tumor size alone is also a significant predictive factor for locoregional failure after TEM for T1 rectal cancer. Local recurrence rates at 3 years for T1 tumors with diameter ≤3 cm were significantly lower [34]. In our results all patients with recurrence had an initial tumor size >5 cm. Also in two of four patients with recurrence only a partial excision was performed.

Even though the recurrence rate in our collective is relatively high (9.8%), the rate of failure observed in this collective was 2.4%. This observation implies that 97.6% of the study patients remained recurrence free or were successfully treated after relapse. These data demonstrate that TEM is a curative surgical technique.

After relapse the therapeutic trial with TEM was not a disadvantage with regard to the prognosis in three of four cases. Particularly important in this context are continuous follow-up examinations, because 75% of the patients in this study in whom a relapse was diagnosed early were successfully treated. The different approach in therapy after relapse is caused in different wishes of patients and concomitant diseases. Two patients were given radiochemotherapy and re-TEM after relapse. A radical surgical procedure was rejected by the patients because of multimorbidity.

Taking these data and the reduced surgical trauma, TEM provides an adequate primary method for treatment in this tumor stage. This finding is consistent with the experiences described by other authors for treatment of early rectal cancer [12,21]. Moreover, the fact that patients who suffer a relapse can still be successfully treated is also described in the literature [21]. Nevertheless, the indication spectrum of TEM in clinical daily routine has become smaller since the introduction of ESD.

## Conclusions

Our results confirm that TEM is an appropriate method for treating broad-based rectal adenomas. For T1 low-risk carcinomas TEM offers a therapeutic option with low complication rates and a low rate of therapeutic failure.

## Abbreviations

ESD: Endoscopic Submucosal Dissection; TEM: Transanal Endoscopic Microsurgery.

## Competing interests

The authors declare that they have no competing interests.

## Authors' contributions

MA, GFB, JB, and AM were responsible for the study concept and design. MA and AM were responsible for data acquisition and conception of the manuscript. MA, AM, and AK were responsible for statistical analysis. All authors were responsible for data analysis and interpretation. MA and AM drafted and designed the manuscript and contributed equally to this work. MA, AM, and AK were responsible for manuscript preparation. All authors reviewed the manuscript. All authors read and approved the final manuscript.
